# Celestial Bodies Far-Range Detection with Deep-Space CubeSats

**DOI:** 10.3390/s23094544

**Published:** 2023-05-07

**Authors:** Vittorio Franzese, Francesco Topputo

**Affiliations:** Department of Aerospace Science and Technology, Politecnico di Milano, Via La Masa, 34, 20156 Milano, Italy; francesco.topputo@polimi.it

**Keywords:** celestial bodies, asteroids, far-range detection, CubeSats, deep space

## Abstract

Detecting celestial bodies while in deep-space travel is a critical task for the correct execution of space missions. Major bodies such as planets are bright and therefore easy to observe, while small bodies can be faint and therefore difficult to observe. A critical task for both rendezvous and fly-by missions is to detect asteroid targets, either for relative navigation or for opportunistic observations. Traditional, large spacecraft missions can detect small bodies from far away, owing to the large aperture of the onboard optical cameras. This is not the case for deep-space miniaturized satellites, whose small-aperture cameras pose new challenges in detecting and tracking the line-of-sight directions to small bodies. This paper investigates the celestial bodies far-range detection limits for deep-space CubeSats, suggesting active measures for small bodies detection. The M–ARGO CubeSat mission is considered as the study case for this activity. The analyses show that the detection of small asteroids (with absolute magnitude fainter than 24) is expected to be in the range of 30,000–50,000 km, exploiting typical miniaturized cameras for deep-space CubeSats. Given the limited detection range, this paper recommends to include a zero-phase-angle way point at close range in the mission design phase of asteroid rendezvous missions exploiting deep-space CubeSats to allow detection.

## 1. Introduction

Asteroid rendezvous and fly-by missions are becoming more and more frequent owing to the interest of the scientific community in small bodies [1]. Asteroids, comets, and minor planets differ each other and are therefore all appealing targets for a deeper understanding of the history of the solar system. Out of more than one million minor bodies in the solar system (see https://www.minorplanetcenter.net/iau/mpc.html (accessed on 19 January 2023)), only a few have been closely observed by spacecraft since a dedicated mission can visit only one or a few more objects in a few years. Missions such as ROSETTA [2], Hayabusa [3], Hayabusa 2 [4], OSIRIS-REx [5], and DART [6] have visited comets and asteroids from a close distance, providing valuable information on the bodies’ physical and morphological properties, which are not deducible from ground-based observations. During the deep-space travel, these missions detect the small body target from optical cameras and track the relative line-of-sight (LoS) direction in time [7]. This is needed to feed relative estimation algorithms and lock the target in the spacecraft sensors field of view, therefore assuring reaching the final destination. The large aperture cameras of conventional spacecraft allow far-range asteroid detection, even at distances of 0.5 AU [8] due to the considerable amount of light that can be acquired from these sensors [9].

The modern trend in the space sector is to scale down the spacecraft components and platforms while still retaining similar mission objectives of traditional missions [10]. Nanosatellites such as CubeSats [11] are miniaturized platforms constituted by modular units (1U is a cube of 10 cm edge), which have reduced the entry-level cost of space missions, being assembled using commercial off-the-shelf (COTS) components and launched in space in a ride-share configuration as in the Artemis-1 mission [12]. The CubeSat specifications were first developed for teaching and demonstration purposes in 1999 [13]. Then, CubeSats became an asset for private companies too, being ideal platforms for technology demonstration. CubeSats are now a standard platform considered for missions in the proximity of Earth.

Small satellites will soon invade deep space [14] after the success of the first deep-space CubeSat mission, MarCO [15]. The European Space Agency has promoted many deep-space CubeSat studies, such as M–ARGO for asteroid rendezvouz [16], LUMIO for lunar observation [17,18], and CubeSats to be released in the Didymos asteroid environment by the Hera mothercraft [19] (Milani [20] and Juventas [21]). NASA has funded many SmallSat mission that flew along the Artemis-1 mission: these comprised NEA-Scout [22], ArgoMoon [23], BioSentinel [24], and others. In addition, JAXA is adopting deep-space CubeSats as manifested by the EQUULEUS mission case [25].

The far-range detection of asteroids exploiting miniaturized components is a critical task for deep-space CubeSats, as the small aperture and the related low-limit magnitude of the onboard sensor do not allow much light to be received and processed onboard. This is a significant issue if we consider the relatively large uncertainties on the spacecraft and asteroid positions, which raises questions on the overall feasibility of small-body missions implementing nanosatellites. To date, no deep-space CubeSat missions have ever explored very small and dim asteroids (estimated diameter lower than 50 m), and the only functioning CubeSat for asteroid observation ever flown to date is LICIACube, whose asteroid target, Didymos, is a large asteroid (780 m diameter) and therefore quite bright.

The contributions of this paper are (1) to derive the celestial bodies detectability conditions in view of miniaturized sensors for deep-space CubeSats, (2) to determine the ranges and phase angles figures that allow planets and asteroids detection for deep-space CubeSats, and (3) to provide recommendations for small-body missions to be considered already at the mission design stage in the case of miniaturized platform and sensors. The Miniaturized Asteroid Remote Geophysical Observer (M–ARGO), a typical deep-space CubeSat mission, is considered as the use case.

This paper is structured as follows. Section 2 details the magnitude model, Section 3 describes the radiometric model for celestial bodies, Section 4 derives the signal to noise model, and Section 5 derives the detectability conditions and envelope of celestial bodies for deep-space CubeSats. Finally, concluding remarks are given in Section 6.

## 2. Absolute and Apparent Magnitude Models

The detection of a celestial object is related to the capability of distinguishing its signal among other sources. The signal coming from objects in the solar systems is the reflected light from the sun, and thus it is a function of the observer–object geometry (distance and phase angle) and the object properties (e.g., albedo). The best observation scenario is when the object is in opposition, that is, when the observer is placed between the sun and the object. The major objects in the solar system (planets) can be observed even with the naked eye because they are big and have a high albedo, while minor bodies (e.g., asteroids) are very faint due to their small size. Technically, the definition of bright and faint sources is always with respect to the capability of detecting them. Thus, this section elaborates on the radiometric models adopted to detect minor bodies.

### 2.1. Magnitude Scale

The signal coming from a source is actually a power flux collected by a given area. The light reflected by the source in all the directions decreases with the distance of a factor proportional to $r^{-2}$, where *r* is the relative distance. This effect is known as spherical loss. In reality, it is not a loss of power, but actually the overall reflected power is spread across always bigger spheres as the distance increases. Now, astronomers have created a magnitude model which relates the power fluxes to reference values. Calling *F* the power flux, the equation which relates the flux *F* to the magnitude *m* is [26]

(1) $$\frac{F}{F_{\mathrm{ref}}}=10^{-0.4(m-m_{\mathrm{ref}})}$$

where $F_{\mathrm{ref}}$ is a reference flux, and $m_{\mathrm{ref}}$ is its magnitude. Note that the flux is given in ${\mathrm{Wm}}^{-2}$. As a reference, the sun has a magnitude of $m_{S}=-26.74$ as seen from the earth and a corresponding flux of $F_{S}=1367$ ${\mathrm{Wm}}^{-2}$. Thus, any flux can be converted into magnitude by using as reference values the ones from the sun. The magnitude scale is an inverse scale, which means that a source brighter than another one has a magnitude lower than the other.

### 2.2. Apparent Magnitude of Major Bodies

**Figure 1:**
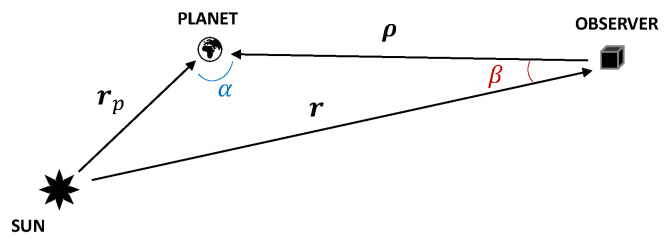
Observation geometry for the planets’ apparent magnitude assessment.

The major bodies in the solar system vary in size, composition, albedo, and distance to the sun. The astronomic community created a unified model from empirical data to derive the apparent magnitude of a planet as a function of the observation geometry. Figure 1 shows the observer–object relative geometry. The planet position vector with respect to the observer is denoted with ρ, the planet position with respect to the sun with $r_{p}$, the planet phase angle as seen from the observer with α, and the sun aspect angle as seen from the observer pointing to the planet with β.

Now, denoting with ρ and $r_{p}$ the respective moduli, the apparent magnitude *V* of a planet can be expressed as [26]

(2) $$V=V\;\left(1,0\right)+5\;\log_{10}\;\left(\rho \;r_{p}\right)+m$$

where V(1,0) is the apparent magnitude of the planet at 1 AU from the sun and at 0 phase angle (also called planet absolute magnitude) and *m* the phase law. The distances are expressed in AU. The planet’s absolute magnitude and phase law (determined from observations) have been retrieved from [26].

### 2.3. Absolute Magnitude of Minor Bodies

The vast majority of minor bodies in the solar systems are observed from ground antennas. Commonly, the antennas point to a region of sky in sidereal tracking (thus, following the stars) to gather pictures. In this way, if an asteroid is passing by that region, it will be seen as a moving light dot. Upon confirmation, the asteroid can be detected and its ephemeris determined according to the relative motion during the observation window. The asteroid ephemerides are then refined with successive observations. All the information regarding the minor bodies are collected in the Minor Planet Center (MPC) (see https://www.minorplanetcenter.net/iau/mpc.html (accessed on 19 January 2023); this research has made use of data and/or services provided by the International Astronomical Union’s Minor Planet Center), which is responsible for gathering and managing the full list of known minor bodies in the solar system. The direct information that can be observed by an asteroid is its light curve, which is the time evolution of the light received from an asteroid during the observation. The repetitive pattern of the light curve gives information about the asteroid rotational period, while the apparent magnitude coupled with the orbital information yields information on the object size [27]. Indeed, the asteroids are modeled as spheres that reflect sunlight in space. So, considering the amount of light received by a sensor, the distance to the target, its phase angle with the sun, and an assumed albedo, the amount of light radiated in space is proportional to the size of the asteroid, which can be then determined.

**Figure 2:**
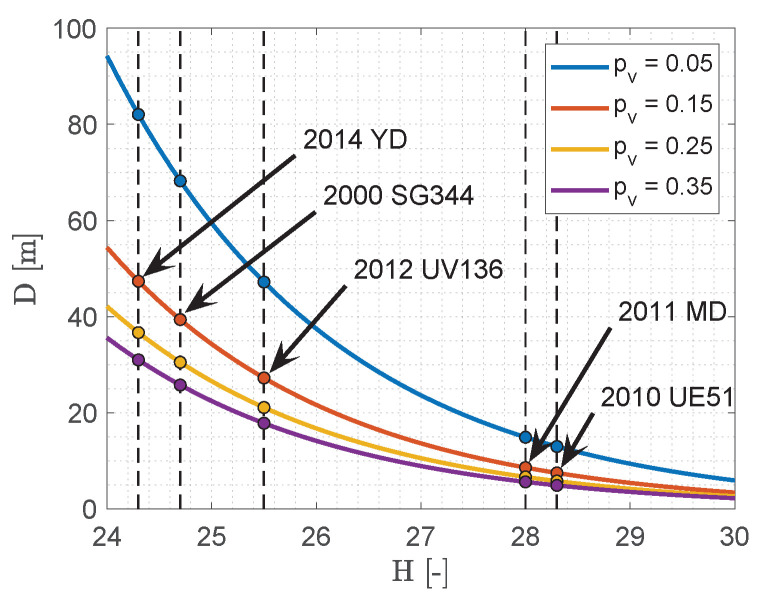
Albedo and diameter uncertainties for typical asteroids.

Quite a few asteroids have known geometry, this is why they are commonly assumed to be spherical. The diameter of an asteroid is linked to a unique parameter known as absolute magnitude *H* through the assumed albedo. This relation is [26]

(3) $$D=\frac{1329}{\sqrt{p_{v}}}10^{-0.2\;H}$$

where *D* is the diameter (expressed in km) and $p_{v}$ is the asteroid albedo. The albedo of the asteroids ranges between 0.05 and 0.4. For unknown asteroids, it is assumed to be 0.15. Figure 2 shows the estimated diameter *D* of typical asteroids (2000 SG344, 2010 UE51, 2011 MD, 2012 UV136, 2014 YD) for different levels of albedo $p_{v}$. The reference diameters are obtained from Equation (3) with an assumed albedo of 0.15. Therefore, it is evident to see how uncertain the physical properties of asteroids are, given the light–curve measurements. However, in order to categorize asteroids, their absolute magnitude *H* is commonly used as a reference value from which the asteroid diameter is estimated, according to an assumed albedo. The asteroid absolute magnitude is then used to determine its apparent magnitude according to an observation geometry.

### 2.4. Apparent Magnitude of Minor Bodies

The apparent magnitude model for a minor body is similar to the one of major bodies. Indeed, the absolute magnitude for minor bodies is defined as the apparent magnitude that the object would have at 1 AU from the sun and 0 phase angle, as for major bodies, and denoted with *H*, whereas it is defined as V(1,0) for the major bodies. For minor bodies, the apparent magnitude *V* can be modeled as [26]

(4) $$V=H+5\;\log_{10}\left(\rho \;r_{a}\right)-2.5\;\log_{10}\left[\left(1-G\right)\;\phi_{1}+G\;\phi_{2}\right]$$

where ρ is the object–observer distance, $r_{a}$ is the sun–object distance, *G* is the slope parameter of the object phase curve (determined from observations), and $\phi_{1}$ and $\phi_{2}$ are phase functions (dependent on the phase angle α, expressed in radians). The distances are expressed in AU. The phase functions are [26]

(5) $$\phi_{1}=\exp\;\left[\;-3.33\;\;\tan\;{\left(\alpha /2\right)}^{0.63}\;\right]\;;\;\phi_{2}=\exp\;\left[\;-1.87\;\;\tan\;{\left(\alpha /2\right)}^{1.22}\;\right]$$

### 2.5. Magnitude Screening of Solar System Objects

Solar system objects shine with reflected sunlight. Among others, the object’s size, shape, surface properties, and distance to the sun characterize the amount of sunlight that is reflected in space. The absolute magnitude of planets lies in the range [0, −10], while for minor planets, it lies in the range [30, 0] and even fainter. For this reason, minor planets are more difficult to be observed than planets, and the geometric distances and phase angles play a crucial role in the resulting apparent magnitude. This is particularly true for optical sensors onboard CubeSats, whose reduced aperture compromises the limit magnitude of the sensor around a value of 6. Regarding minor planets, they are best observed in opposition (α=0) and in proximity.

## 3. Radiometric Model

Section 2 dealt with the magnitude modeling of sources. This section deals with the modeling of the emitted power and photons from a radiating source, which are then reflected from asteroids and acquired by spacecraft sensors.

### 3.1. Irradiance and Flux of Photons

Consider Planck’s law

(6) $$b_{\lambda }=\frac{2\;h\;c^{2}}{\lambda^{5}}\;\frac{1}{e^{\frac{hc}{\lambda k_{B}T}}-1}$$

where *h* is Planck’s constant, *c* the speed of light, λ the wavelength, $k_{B}$ the Boltzmann’s constant, and *T* the temperature. The function $b_{\lambda }$ is the spectral distribution of electromagnetic radiation emitted by a black body at a given temperature *T*, and it is given in $W\;m^{-3}{\mathrm{sr}}^{-1}$. Thus, it models the amount of energy emitted by a source per unit time, per unit area normal to the propagation, per unit solid angle, and per unit wavelength. The function $b_{\lambda }$ is also known as irradiance. It is possible to convert from the irradiance information to the spectral distribution of emitted photons considering that (1) the frequency ν and the wavelength λ of the photons are related by the speed of light as *c* = νλ, and (2) the energy of a photon at a given wavelength is given by Planck’s relation $E_{\lambda }$ = hν. The spectral distribution of photons emitted by a radiating source can be obtained by dividing Planck’s law by the energy of a single photon as

(7) $$p_{\lambda }=\frac{b_{\lambda }}{E_{\lambda }}=\frac{2\;c}{\lambda^{4}}\;\frac{1}{e^{\frac{hc}{\lambda k_{B}T}}-1}$$

The units of measure of $p_{\lambda }$ are $s^{-1}m^{-3}{\mathrm{sr}}^{-1}$. The function $p_{\lambda }$ models the number of photons emitted by a source per unit surface, solid angle, wavelength, and time.

### 3.2. Emitted Fluxes in a Frequency Range

The power flux and the flux of photons in a given spectral range can be obtained by integrating $b_{\lambda }$ and $p_{\lambda }$ in the frequency range of interest, respectively. The sun can be considered a black-body radiator having a temperature *T* = 5778 K. The power flux emitted by the sun in a given spectral range can be obtained integrating Equation (6) between lower and upper wavelength bounds ($\lambda_{L}$ and $\lambda_{U}$, respectively) as

(8) $$B=\int_{\lambda_{L}}^{\lambda_{U}}b_{\lambda }\;d\lambda =\int_{\lambda_{L}}^{\lambda_{U}}\frac{2\;h\;c^{2}}{\lambda^{5}}\;\frac{1}{e^{\frac{hc}{\lambda k_{B}T}}-1}d\lambda$$

Integrating Equation (8) between 0 and +∞ yields the emitted energy by the sun per unit area, unit solid angle, and time. This integral yields 2.0130 × $10^{7}$ watts per unit surface and solid angle emitted by the sun overall the whole spectrum. Considering the visible band ($\lambda_{L}$ = 380 nm, $\lambda_{U}$ = 740 nm), the sun emits 8.6299 × $10^{6}$ watts per unit surface and solid angle. Thus, 42.87% of the radiation emitted by the sun lies in the visible spectrum.

The flux of photons in a given wavelengths range can be obtained by integrating $p_{\lambda }$ between the lower and upper bounds of the frequency range of interest ($\lambda_{L}$ and $\lambda_{U}$). Thus, the flux of photons in a given frequency range (*P*) can be obtained as

(9) $$P=\int_{\lambda_{L}}^{\lambda_{U}}p_{\lambda }\;d\lambda =\int_{\lambda_{L}}^{\lambda_{U}}\frac{2\;c}{\lambda^{4}}\;\frac{1}{e^{\frac{hc}{\lambda k_{B}T}}-1}d\lambda$$

The amount of photons ejected in the visible band can be computed by evaluating the integral in Equation (9) with $\lambda_{L}$ = 380 nm and $\lambda_{U}$ = 740 nm, which are the bounds of the visible spectrum. This results in 2.4086 × $10^{25}$ photons emitted in the visible spectrum per unit time, unit area normal to the propagation, and unit solid angle. Evaluating the integral from 0 to +∞ yields 9.3359 × $10^{25}$ photons emitted overall in the whole spectrum per unit time, unit area, and unit solid angle. Thus, 25.77% of the overall photons emitted by the sun belong to the frequencies in the visible spectrum.

### 3.3. Overall Emitted Quantities

The luminosity of the sun *L*, defined as the energy emitted in all the directions per unit time, is given by the integral of the spectral emission across all the wavelengths multiplied by π (isotropic radiation) and for the external area of the sun. The external area of the sun is $4\pi r_{s}^{2}$, where $r_{s}$ is the sun radius. Note that the overall luminosity *L* can be computed by considering directly the emitted power of a spherical source through the Stefan–Boltzmann law multiplied by the external area of a sphere. Thus *L* can be computed with any of the following two:

(10) $$L=\pi \;\left(4\pi r_{s}^{2}\right)\;\int_{0}^{+\infty }b_{\lambda }\;d\lambda \;\;L=\sigma T^{4}\;\left(4\pi r_{s}^{2}\right)$$

where $\sigma =5.67^{-8}$ $Wm^{-2}K^{-4}$ is the Stefan–Boltzmann constant. The luminosity of the sun in the visible spectrum $L_{V}$ can be obtained by delimiting the integration in Equation (10) to the visible band:

(11) $$L_{V}=\pi \;\left(4\pi r_{s}^{2}\right)\;\int_{\lambda_{L}}^{\lambda_{U}}b_{\lambda }\;d\lambda$$

The same reasoning applies to the number of emitted photons. Thus, the overall emitted photons (*J*) and the emitted photons in the visible band ($J_{V}$) are given by

(12) $$J=\pi \;\left(4\pi r_{s}^{2}\right)\;\int_{0}^{+\infty }p_{\lambda }\;d\lambda \;\;J_{V}=\pi \;\left(4\pi r_{s}^{2}\right)\;\int_{\lambda_{L}}^{\lambda_{U}}p_{\lambda }\;d\lambda$$

### 3.4. Fluxes

The power emitted by a source goes across all the directions in space. Thus, when the distance to the source increases, the power is spread across always bigger spheres, determining a decrease in the power flux. Denoting as *r* a generic distance to a source, the factor ${\left(4\pi r^{2}\right)}^{-1}$ models the spreadness of a quantity across always bigger spheres with increasing radius. For this reason the power flux decreases when the distance to a source increases. Calling *R* the earth’s distance to the sun, the power flux received at the earth can be obtained as

(13) $$F=\frac{1}{4\pi R^{2}}\;\pi \;\left(4\pi r_{s}^{2}\right)\;\int_{0}^{+\infty }b_{\lambda }d\lambda \;\to \;F=\sigma T^{4}\frac{4\pi r_{s}^{2}}{4\pi R^{2}}$$

The flux received at the earth considering the whole spectrum can thus be obtained using Equation (13), and its value is 1366 W/m^2^ (this is also known as the solar constant). Similarly, the power flux at the earth’s location from the sun in the visible band is

(14) $$F_{V}=\frac{1}{4\pi R^{2}}\;\pi \;\left(4\pi r_{s}^{2}\right)\;\int_{\lambda_{L}}^{\lambda_{U}}b_{\lambda }\;d\lambda =585.5\;W/m^{2}$$

with $\lambda_{L}$ = 380 nm and $\lambda_{U}$ = 740 nm.

The flux of photons can be derived in a similar way. The overall photons flux at the earth location (*G*) and the photons flux in the visible band ($G_{V}$) are, respectively,

(15) $$G=\frac{1}{4\pi R^{2}}\;\pi \;\left(4\pi r_{s}^{2}\right)\;\int_{0}^{+\infty }p_{\lambda }\;d\lambda \;\;G_{V}=\frac{1}{4\pi R^{2}}\;\pi \;\left(4\pi r_{s}^{2}\right)\;\int_{\lambda_{L}}^{\lambda_{U}}p_{\lambda }\;d\lambda$$

### 3.5. Fluxes from an Asteroid

The modeling of the radiating sources and related fluxes serves as inputs to model the fluxes coming from asteroids. In particular, the logical procedure foresees two steps, which are detailed in the following:

1. Let us consider the irradiated power from the sun across all directions. At a given distance, an asteroid receives an incoming power flux from the sun. The overall incident power on the asteroid is given by the power flux from the sun multiplied by the exposed surface of the asteroid, which can be modeled as a half-sphere. Therefore, the power flux in the visible spectrum from the sun at the asteroid location is easily modeled as
  (16) $$F_{V}=\frac{1}{4\pi R_{a}^{2}}\;\pi \;\left(4\pi r_{s}^{2}\right)\;\int_{\lambda_{L}}^{\lambda_{U}}\frac{2h\;c^{2}}{\lambda^{5}}\;\frac{1}{e^{\frac{hc}{\lambda k_{B}T}}-1}d\lambda$$
  where $R_{a}$ is the asteroid distance to the sun.
  Quite a few asteroids have known geometry, and thus they are assumed to be spherical with a diameter given by Equation (3). The albedo of the asteroids ranges between 0.05 and 0.4, and it is usually assumed to be 0.15 for unknown asteroids. Thus, the power received at the asteroid is the incident flux (Equation (16)) multiplied by $\pi D^{2}/2$ (the external surface of an half sphere).
2. Part of the incident power on the asteroid in the visible band is reflected back into space according to its albedo $p_{v}$. An observer placed at a given distance $R_{o}$ to the asteroid which sees the asteroid with a phase angle α will receive an incoming power flux of
  (17) $$F_{V}=\pi \int_{\lambda_{L}}^{\lambda_{U}}\frac{2\;h\;c^{2}}{\lambda^{5}}\;\frac{1}{e^{\frac{hc}{\lambda k_{B}T}}-1}d\lambda \;\left(4\pi r_{s}^{2}\right)\;\frac{1}{4\pi R_{a}^{2}}\;p_{v}\;\left(\pi D^{2}/2\right)\frac{1}{4\pi R_{o}^{2}}\frac{\cos\alpha +1}{2}$$
  where the term (cosα+1)/2 models the decrease in power as a function of the asteroid phase angle α. This term is 1 at 0 deg phase angle, 1/2 at 90 deg, and 0 at 180 deg. The same procedure can be adopted to determine the received photons flux in the visible band from an asteroid. It can be obtained by dividing Equation (17) by the energy of a photon. Thus,
  (18) $$G_{V}=\pi \int_{\lambda_{L}}^{\lambda_{U}}\frac{2\;c}{\lambda^{4}}\;\frac{1}{e^{\frac{hc}{\lambda k_{B}T}}-1}d\lambda \;\left(4\pi r_{s}^{2}\right)\;\frac{1}{4\pi R_{a}^{2}}\;p_{v}\;\left(\pi D^{2}/2\right)\frac{1}{4\pi R_{o}^{2}}\frac{\cos\alpha +1}{2}$$
  These power and photon fluxes models will be used to derive the signal-to-noise ratios of the asteroids during a spacecraft acquisition.

## 4. Signal to Noise Ratio

The radiometric model shown in Section 3 permits to derive the strength of an object signal with respect to the other sources, considering the relative geometry and the camera characteristics. This is the signal-to-noise ratio (SNR). The signal-to-noise ratio is defined according to the photons coming from a given source with respect to the noise floor, which is constituted by the standard deviation of all the photons, which are not signals.

### 4.1. Signal Model

The signal collected from a camera sensor is proportional to the number of collected photons. Thus, the signal is proportional to the incident flux $G_{s}$, the aperture area *A*, and the exposure time $t_{exp}$. Once entered in the entrance pupil, the photons pass through the camera optics, where some of the photons are absorbed and some others are reflected. The optic lens reduction factor $\xi_{r}$ is a property of the camera optics and describes the ratio between the photons that pass through the optics to the overall incoming photons. In this way, it can be seen as the fraction of photons that are not reflected nor absorbed by the optics. Then, after the optics, the photons are collected by a light sensor (e.g., a CCD) and converted into electrons. Every light sensor has a certain quantum efficiency (QE) that is defined as the ratio between the detected photons (converted into electrons) and the overall incoming photons. Most of the CCDs have a mean quantum efficiency ($\eta_{qe}$) over the visible band of 0.7, or even higher. Thus, the number of photons coming from an asteroid, collected by a CCD sensor, and converted into electron counts in a given time window can be modeled as

(19) $$S_{s}=\underset{G_{s}}{\underbrace{\pi \int_{\lambda_{L}}^{\lambda_{U}}\frac{2\;c}{\lambda^{4}}\;\frac{1}{e^{\frac{hc}{\lambda k_{B}T}}-1}d\lambda \;\left(4\pi r_{s}^{2}\right)p_{v}\;\left(\pi D^{2}/2\right)\frac{1}{4\pi R_{a}^{2}}\frac{1}{4\pi R_{o}^{2}}\frac{\cos\alpha +1}{2}}}\;\xi_{r}\;\eta_{qe}\;A\;t_{exp}$$

So, $S_{s}$ constitutes the signal from a given source. Note that in Equation (19), all the quantities related to the orbital geometry, the emitting source, the reflecting source, and the instrument can be found. The quantities which are a function of the orbital geometry are the spherical losses and the phase angle loss, the quantities belonging to the asteroid are its albedo and size, the quantity related to the emitting source is the emitted flux, and the quantities related to the instrument are the reduction factor, the quantum efficiency, the collecting area, and the exposure time.

### 4.2. Noise Model

The noise taking part in the measurements of a signal are presented in this section. Note that the signal is expressed in $e^{-}$, while the noise is expressed in $\sqrt{e^{-}}$ (Poisson process).

**Signal shot noise**. The noise due to the source itself (called signal shot noise $N_{s}$) is given by the standard deviation of the source signal itself. Thus,

(20) $$N_{s}=\sqrt{S_{s}}$$

**Background noise**. The photons coming from the background sky are modeled as a mean cosmic background flux $G_{\mathrm{sky}}$ multiplied by the collecting area, the exposure time, and the instrumentation efficiencies. Thus, the noise associated to the sky is modeled as

(21) $$N_{\mathrm{sky}}=\sqrt{G_{\mathrm{sky}}\;\xi_{r}\;\eta_{\mathrm{qe}}\;A\;t_{\exp}}$$

**Read-out noise**. The read-out noise is a byproduct of the reading of the photons from the detector. This is commonly assumed to have a constant amplitude $N_{\mathrm{rn}}$.

**Quantization noise**. The quantization noise is due to the analog-to-digital conversion which has a finite number of bits to represent the signal. The noise can be modeled as

(22) $$N_{q}=\sqrt{\frac{\mathrm{FWC}}{2^{nb}\sqrt{12}}}$$

where FWC is the full-well charge of the detector, which is the maximum charge that can be collected on a single pixel, and nb the number of bits.

**Photo response non-uniformity**. The photo response non-uniformity models the different response of each pixel to the number of incoming photons. Indeed, given the same amount of photons, the number of collected electrons is not the same for every pixel due to small imperfections. Thus, this non-uniformity can be modeled as

(23) $$N_{\mathrm{prnu}}=\sqrt{pS_{s}}$$

where *p* is the PRNU factor and $S_{s}$ is the signal.

**Dark current**. The dark current noise is generated by the thermal energy of the CCD. The mean level of the dark current can be easily subtracted from each image, but the dark current shot noise (DCSN) will be present. This is modeled as

(24) $$N_{\mathrm{dcsn}}=\sqrt{\Phi_{\mathrm{dcsn}}t_{\exp}}$$

where $\Phi_{\mathrm{dcsn}}$ is the dark current shot noise flux (in $e^{-}$/s).

**Fixed pattern noise**. The fixed pattern noise (FPN) is an offset which is constant in time but variable in space (over the detector). It is made up of three components: per pixel FPN, per-column FPN, and per-row FPN. These noise sources can be modeled as having maximum noise values of $N_{\mathrm{pfpn}}$, $N_{\mathrm{cfpn}}$, and $N_{\mathrm{rfpn}}$, in units of $\sqrt{e^{-}}$.

### 4.3. SNR

The signal-to-noise ratio (SNR) is a measure of how much a signal from a source is strong with respect to the overall noise. Bright sources have high SNR, whereas faint sources are difficult to be detected due to their low SNR. The signal-to-noise ratio is thus defined as the ratio between the number of detected photons from a source $S_{s}$ divided by the standard deviation of all the incoming photons $S_{n}$, that is

(25) $$\mathrm{SNR}=\frac{S_{s}}{\sqrt{S_{n}}}=\frac{S_{s}}{\sqrt{N_{s}^{2}+N_{\mathrm{sky}}^{2}+N_{\mathrm{rn}}^{2}+N_{q}^{2}+N_{\mathrm{prnu}}^{2}+N_{\mathrm{dcsn}}^{2}+N_{\mathrm{dcnu}}^{2}+N_{\mathrm{fpn}}^{2}+N_{\mathrm{cfpn}}^{2}+N_{\mathrm{rfpn}}^{2}}}.$$

## 5. Celestial Bodies Far-Range Detection

This section now deals with the assessment of the detection limits of celestial bodies, given the observer and target properties and relative geometry. In order to assess the performance in a typical deep-space miniaturized spacecraft mission, the M–ARGO mission is considered as the use case, and the detection of celestial objects is performed in this context.

### 5.1. The M–ARGO Mission

The Miniaturized Asteroid Remote Geophysical Observer (M–ARGO) is a 12 U deep-space CubeSat that is planned to piggyback on the launch of another large spacecraft going toward the sun–earth Lagrange point $L_{2}$. After insertion into a halo parking orbit at $L_{2}$, M–ARGO will depart from there, performing a deep-space cruise toward a NEA target using low-thrust electric propulsion [14]. The maximum transfer time to the asteroid is set to 3 years, and the duration of close-proximity operations (CPO) is planned to last up to 6 months. The objective of M–ARGO is to characterize the physical properties of the target NEA for the presence of in situ resources. The preliminary spacecraft mass budget amounts to 22.6 kg, where 2.8 kg accounts for the propellant. Table 1 presents a summary of the M–ARGO characteristics. Further details on the M–ARGO mission are present in [28].

**Figure 3:**
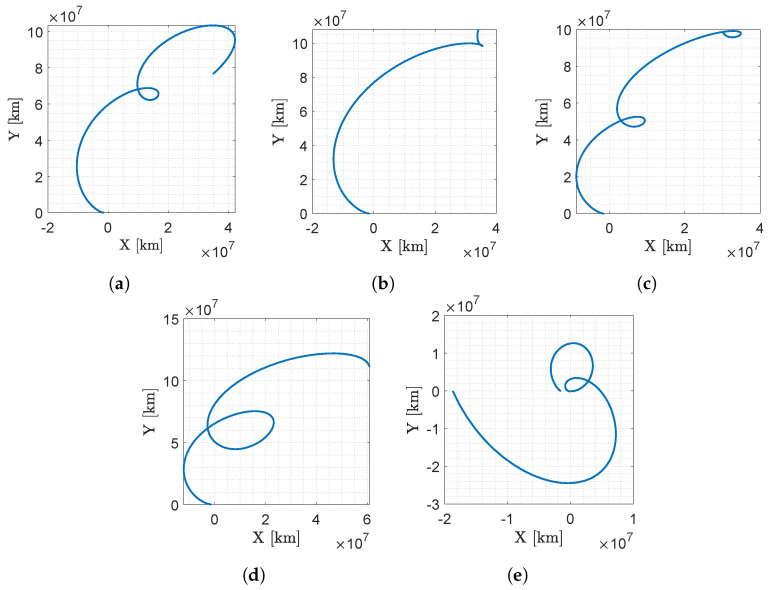
M–ARGO baseline trajectories to the five selected asteroids in the GSE frame. The earth is at (0, 0), the sun is always at (−1, 0) direction, the M–ARGO departure is from the sun–earth L2 point and the asteroid position at arrival is at the final location of the trajectory. (**a**) 2000 SG344; (**b**) 2010 UE51; (**c**) 2011 MD; (**d**) 2012 UV136; (**e**) 2014 YD.

The preliminary five near-Earth asteroids selected for M–ARGO are 2000 SG344, 2010 UE51, 2011 MD, 2012 UV136, and 2014 YD. Time- and fuel-optimal trajectories using electrical low-thrust propulsion are computed starting from the sun–earth L_2_ point to the asteroids. The baseline trajectories for the five selected asteroids are shown in Figure 3. The trajectories are shown in the geocentric solar ecliptic (GSE) frame that originates in the earth; the x axis points toward the sun; the z axis is parallel to the ecliptic north pole; and its y axis completes the right-handed frame. Table 2 reports the orbital elements of the five target asteroids together with their absolute magnitude H and estimated diameter D.

### 5.2. Apparent Magnitude Assessment

**Figure 4:**
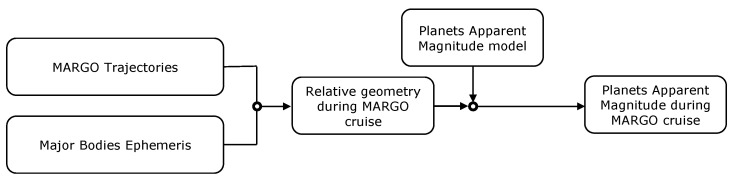
Flow chart of planets’ apparent magnitude assessment.

**Figure 5:**
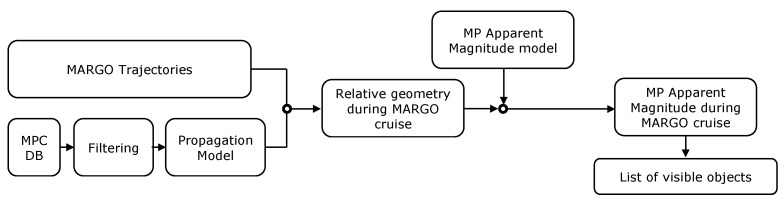
Flow chart of minor planets’ apparent magnitude assessment.

The apparent magnitude of planets and minor bodies as seen from M–ARGO can now be assessed. Figure 4 and Figure 5 show the flow charts used to assess the objects’ magnitude. Regarding the major bodies (Figure 4), their ephemerides were obtained through the SPICE toolkit [29] in the same epochs of the M–ARGO trajectories to determine the relative geometry (distances and phase angles). This geometry is the input to the planets’ apparent magnitude model (Section 2.2) to determine their apparent magnitude for M–ARGO.

Regarding the minor planets, the database of all the objects was retrieved from the Minor Planet Center, which was pre-filtered by selecting only the objects observed more than 80 times to have an higher accuracy in their ephemeris. The orbits of these objects were propagated with a simple two-body problem; then the relative geometry between M–ARGO and the objects was obtained. So, the apparent magnitude model for minor bodies (Section 2.4) was used to retrieve the list of visible targets according to the given camera performance.

### 5.3. Planets Apparent Magnitude

**Figure 6:**
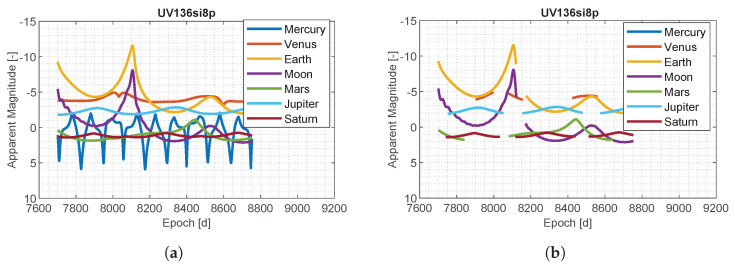
Planets app. magnitude during M–ARGO trajectories (30 deg sun angle). (**a**) M–ARGO to UV136; (**b**) M–ARGO to UV136 (30 deg sun angle).

The apparent magnitude of planets as a function of M–ARGO to UV136 is shown in Figure 6a. Mercury spans between mag 5 and −3, Venus is approximately stable around −5, the earth has a mag always lower than −2, the moon has a similar trend to that of the earth but with five more levels of magnitude, Mars spans between 3 and −3, Jupiter is approximately stable around mag −3, and Saturn oscillates around magnitude 1. Figure 6b shows the constraints of a 40 deg sun exclusion angle on the planets visibility. In such a case, Mercury is not visible because it is too close to the sun, and Venus is visible for some months, while the other planets experience short periods of conjunction.

### 5.4. Asteroids Apparent Magnitude

The asteroids orbital elements are retrieved from the MPC database, and their orbit is propagated under the two-body problem dynamics. The relative geometry in terms of distances and angles with the M–ARGO trajectories can be then easily computed and given as input to the objects’ apparent magnitude assessment model.

**Figure 7:**
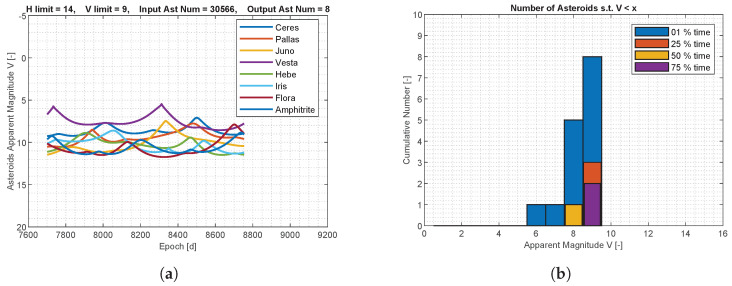
Asteroids’ apparent magnitude for M–ARGO to UV136. (**a**) Asteroids’ apparent magnitude; (**b**) asteroids versus apparent magnitude.

**Figure 8:**
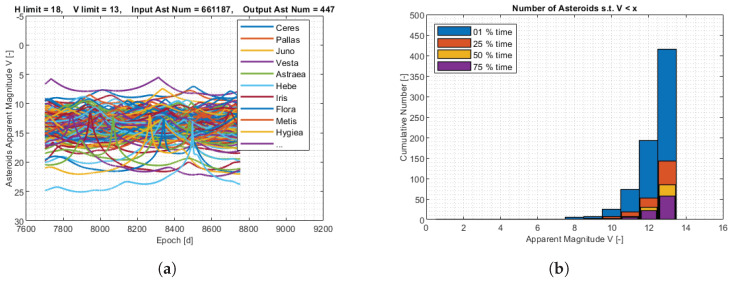
Asteroids’ apparent magnitude for M–ARGO to UV136. (**a**) Asteroids’ apparent magnitude; (**b**) asteroids versus apparent magnitude.

The apparent magnitude of the asteroids for M–ARGO to UV136 is shown in Figure 7. The MPC database was pre-filtered such that only the asteroids with an absolute magnitude lower than 14 are considered, resulting in 30,566 objects. Out of these objects, only a few objects reach an apparent magnitude of 9 for at least a moment during the M–ARGO trajectories and just one object reaches an apparent magnitude of 8 for 50% of the M–ARGO cruising time. A deeper analysis is performed with different filtering parameters. The MPC database is now pre-filtered with only the asteroids that have an absolute magnitude higher than 18, resulting in 661,187 objects, and only those that reach magnitude 13 somewhere during the M–ARGO trajectory are saved. Figure 8 shows the apparent magnitude of these asteroids as a function of the M–ARGO trajectory to UV136. Regarding this trajectory, 85 asteroids reach at least a magnitude of 13, 50% of the time; 30 asteroids reach at least a magnitude of 12, 50% of the time; 7 asteroids reach a magnitude of 11, 50% of the time; 3 asteroids reach a magnitude of 10, 50% of the time; and 1 asteroid reaches a magnitude of 9, 50% of the time.

### 5.5. M–ARGO Targets Detection

The section deals with the limits of detection of the M–ARGO targets.

**Figure 9:**
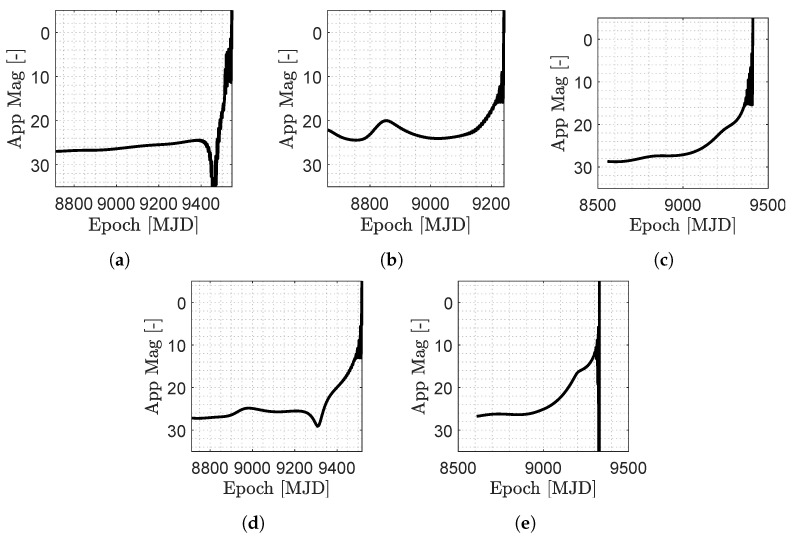
Asteroids apparent magnitude during the M–ARGO baseline trajectories. (**a**) 2000 SG344; (**b**) 2010 UE51; (**c**) 2011 MD; (**d**) 2012 UV136; (**e**) 2014 YD.

**Apparent Magnitude.** The apparent magnitude of the M–ARGO targets (computed with the model in Section 2.4) is shown in Figure 9 for the baseline trajectories. As it can be seen from Figure 9, the apparent magnitude of the M–ARGO targets in deep space is the range 10–30, and they become brighter only when the spacecraft is approaching the targets. Note that typical navcams for CubeSats are revisited versions of the star trackers. Noting that the limit magnitude of common star trackers for CubeSats is in the range 5–7.5 (see Table 3), the M–ARGO targets are not visible for the vast majority of the spacecraft trajectory, and they become visible in the star tracker only during the final approach phase. Table 3 reports the characteristics of current COTS star trackers for CubeSats in terms of the field of view (FOV), camera limit magnitude, acquisition rate, and nominal sun exclusion angle. For CubeSats, the FOV spans between 10 × 12 deg to 15 × 20 deg, the faintest detectable magnitude is 7.5, and the sun exclusion angle spans between 90 and 22 deg according to the proper baffle.

**Figure 10:**
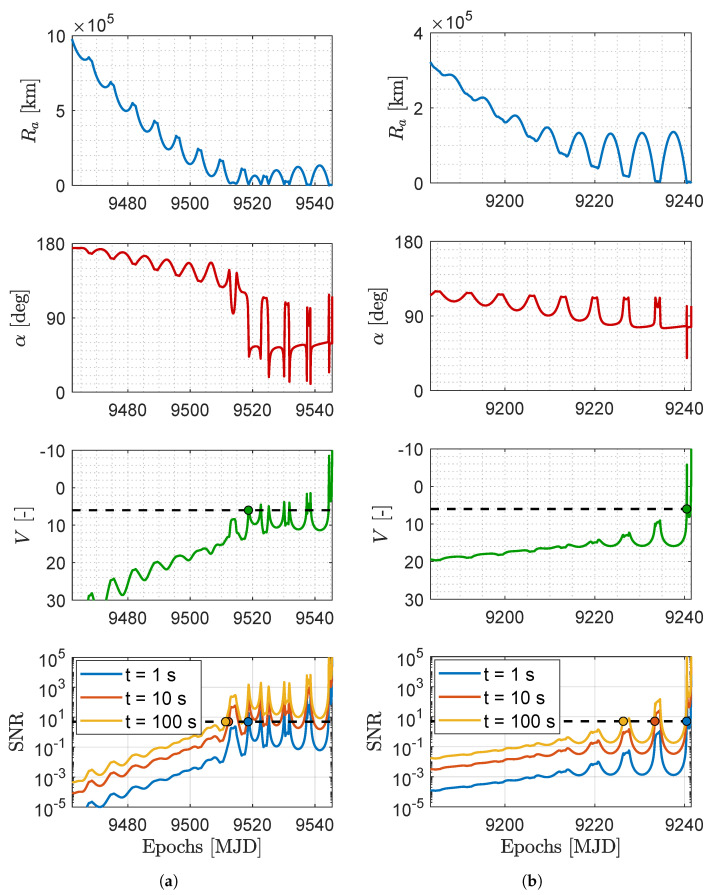
Distance to the asteroid ($R_{a}$), asteroid phase angle (α), asteroid apparent magnitude (*V*), and asteroid SNR for different exposure windows during the M–ARGO arrival at the asteroid (**a**) 2000 SG344 baseline trajectory and (**b**) 2010 UE51 baseline trajectory. The detection epoch (evidenced with a dot) is determined when the apparent magnitude equals 6 (V = 6) or the signal-to-noise ratio equals 5 (SNR = 5).

**Figure 11:**
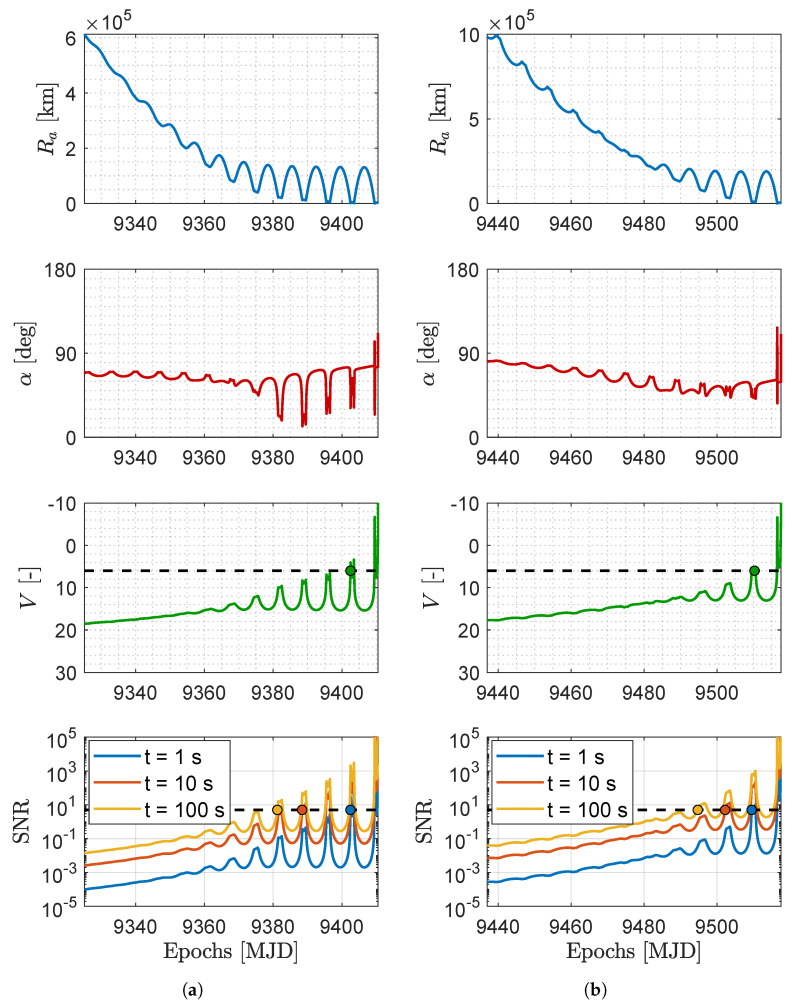
Distance to the asteroid ($R_{a}$), asteroid phase angle (α), asteroid apparent Magnitude (*V*), and asteroid SNR for different exposure windows during the M–ARGO arrival at the asteroid (**a**) 2011 MD baseline trajectory and (**b**) 2012 UV136 baseline trajectory. The detection epoch (evidenced with a dot) is determined when the apparent magnitude equals 6 (V = 6) or the signal-to-noise ratio equals 5 (SNR = 5).

**Signal-to-noise ratio.** The SNR of the asteroids can be evaluated with different exposures to assess their detectability with larger distances. Figure 10 and Figure 11 show the relative geometry and the visibility of the M–ARGO targets for each trajectory. The distance to the target ($R_{a}$), its phase angle (α), its apparent magnitude (*V*), and signal-to-noise ratio (SNR) as seen from M–ARGO are shown in each plot. In terms of apparent magnitude, the detection range is when the sensor limit magnitude is reached (six for common star trackers). Regarding the SNR, a value of five commonly corresponds to a possibility of detection.

The NAVCAM of M–ARGO is taken into account for the SNR during the approach, whose properties are reported in Table 4. Table 5 reports common image sensors considered for the NAVCAM, which are used as a reference to derive the performance of the NAVCAM in Table 6. Thus, the plots in Figure 10 and Figure 11 present the dashed vertical lines in correspondence of a limit magnitude of six and an SNR threshold of five.

Table 7 reports the epochs of the target detection. The first column lists the five different targets, the second column the trajectory, and the third column the arrival epoch. Then, the fourth column shows the detection epoch according to the magnitude model (mag 6), and the remaining columns report the SNR model detections (SNR = 5) with exposures of 1, 10, and 100 s, respectively. The parentheses in the last three columns show the detection epoch difference between the mag and SNR models. There is no difference with 1 s of exposure for the first target detection. Increasing the exposure time to 10 s or 100 s yields a gain of 6–14 days or 7–29 days, respectively, in the epoch of the first detection.

### 5.6. Detectability Envelope

**Figure 12:**
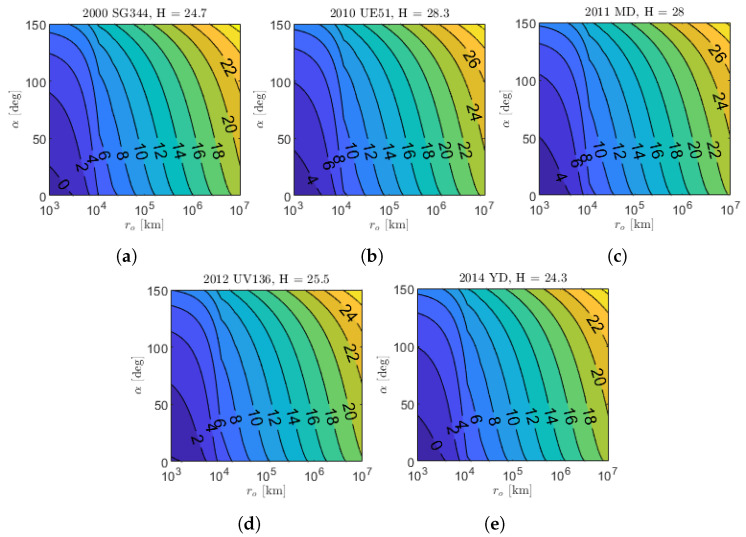
Asteroid app mag function of the relative distance ($r_{o}$) and asteroid phase angle (α). (**a**) 2000 SG344; (**b**) 2010 UE51; (**c**) 2011 MD; (**d**) 2012 UV136; (**e**) 2014 YD.

**Figure 13:**
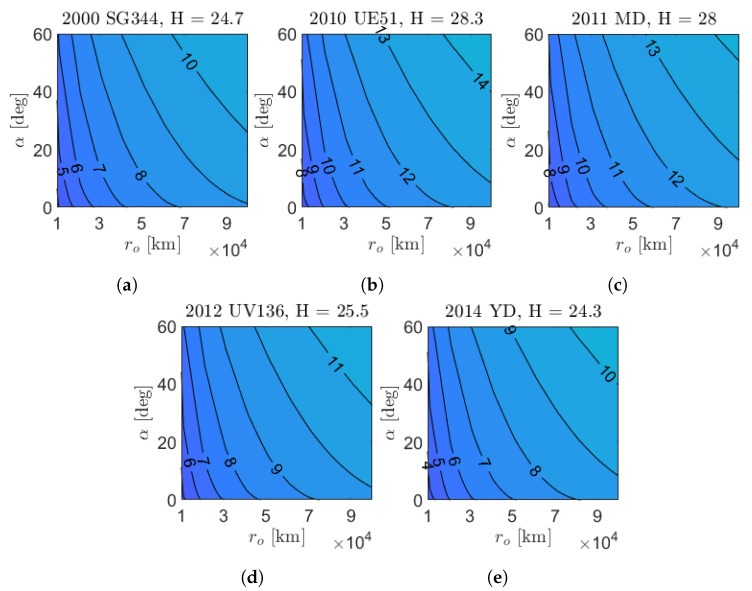
Zoom on the asteroid apparent magnitude in approach conditions. (**a**) 2000 SG344; (**b**) 2010 UE51; (**c**) 2011 MD; (**d**) 2012 UV136; (**e**) 2014 YD.

**Figure 14:**
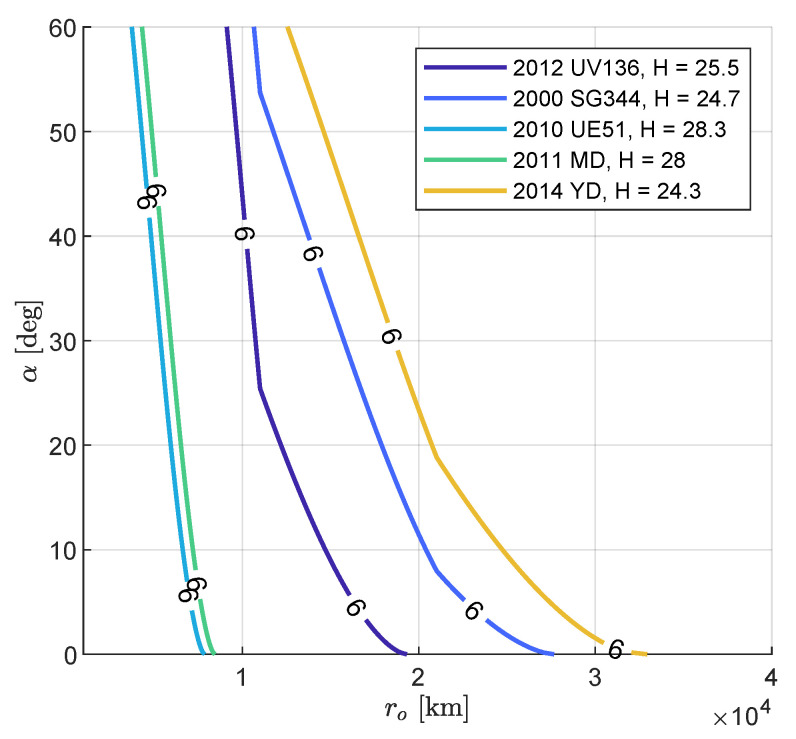
Apparent magnitude 6 for M–ARGO targets as function of range and phase angle.

Figure 12 shows the apparent magnitude contour plots of the five targets for M–ARGO as a function of the asteroid phase angle and relative distance. The asteroids are very small (absolute magnitude H between 24.3 and 28.3); thus, the apparent magnitude six is reached at ranges lower than $10^{5}$ km. A zoom of the same contour plots is shown in Figure 13 for phase angles lower than 60 degrees in the range of $10^{4}$–$10^{5}$ km. Figure 14 shows the detectability envelop of the five M–ARGO targets (that is, the geometry for which they reach mag 6). Clearly, the maximum detectability is reached for phase angles equal to 0. For phase angles lower than 10 degrees, the detectability is expected to be in the range of 30,000–70,000 km, according to the asteroid dimension.

### 5.7. Sensitivity Analysis

This section deals with the analysis of the SNR performance for variations in the camera characteristics, signal, and noise values present in Table 6. In particular, three different categories are set, which are (1) a high-performance camera, (2) a reference camera, and (3) a low-performance camera. The parameters and the corresponding values for the three categories are shown in Table 8.

**Figure 15:**
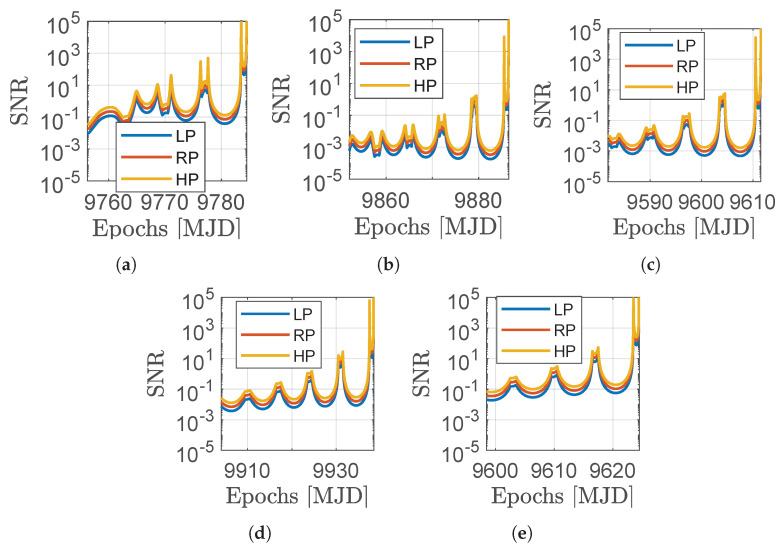
SNR sensitivity analysis in the LP, RP, and HP cases for the five selected targets. (**a**) 2000 SG344; (**b**) 2010 UE51; (**c**) 2011 MD; (**d**) 2012 UV136; (**e**) 2014 YD.

Figure 15 shows the SNR values for the three categories presented in Table 8. A variation in the SNR can be evidenced for the LP, RP, and HP cases; however, the driving factors determining the detectability of the asteorid signal remain the relative distance to the observer and the phase angle, as for dim asteroids, the signal remains rather low.

## 6. Conclusions

Traditional missions are equipped with large aperture cameras, which allow the detection of celestial bodies, even at distances comparable to the astronomical unit. The components for CubeSats are miniaturized versions of large instruments, and the reduced optics yields limited performance in terms of light detectability. In this work, we assessed the detectability limits of planets and asteroids for a deep-space CubeSat mission. The apparent magnitude and the SNR were studied in the context of the M–ARGO mission, including the real spacecraft trajectories, asteroid characteristics, and the miniaturized cameras’ performance in the simulations. For all trajectories, Mercury spans between magnitudes 5 and −3, Venus is approximately stable around −5, the earth has an apparent magnitude always lower than −2, Mars spans between 3 and −3, Jupiter is stable around magnitude −3, and Saturn oscillates around magnitude 1. These values indicate that planets can be detected by deep-space CubeSats with miniaturized sensors. A further analysis on M–ARGO trajectories shows that a total of 85 asteroids reach at least a magnitude of 13, 50% of the time; 7 asteroids reach a magnitude of 11, 50% of the time; and 3 asteroids reach a magnitude of 10, 50% of the time. Recalling that typical optical sensors for CubeSats have a limited magnitude in the range of six, planets can be seen, while asteroids are not visible in deep space. Additionally, the reduced optical performance results limit the detectability range of targets during the approach phase. A radiometric model was developed to evaluate the impact of an increased exposure time of the acquisition on the epoch of target detection. The analyses show that the detection of small asteroids (with absolute magnitude fainter than 24) is expected to be in the range of 30,000–50,000 km, exploiting typical miniaturized cameras for deep-space CubeSats. In essence, when using deep-space CubeSats, the detection of small bodies is challenging; a way point at the zero-phase angle shall be planned already at the mission design phase to detect the target body with a prolonged exposure time.

## Figures and Tables

**Table 1:** M–ARGO characteristics and mission time frame.

Size	Wet Mass	Prop. Mass	Departure (SE L_2_)	Transfer	CPO
12 U	22.6 kg	2.8 kg	2023–2024	≤3 years	≤6 months

**Table 2:** Orbital elements (ecliptic J2000) and properties for the 5 selected asteroids (see https://www.minorplanetcenter.net/iau/mpc.html (accessed on 19 January 2023)).

Name	*a* [AU]	*e* [-]	*i* [deg]	*ω* [deg]	Ω [deg]	*H* [-]	*D* [m]
2000 SG344	0.9775	0.0669	0.1121	275.3026	191.9599	24.7	39.4
2010 UE51	1.0552	0.0597	0.6239	47.2479	32.2993	28.3	7.5
2011 MD	1.0562	0.0371	2.4455	5.9818	271.5986	28	8.6
2012 UV136	1.0073	0.1392	2.2134	288.6071	209.9001	25.5	27.3
2014 YD	1.0721	0.0866	1.7357	34.1161	117.6401	24.3	47.4

**Table 3:** Characteristics of star trackers for CubeSats.

Name	FOV	Limit Mag	Rate	Sun Excl.
Blue C. NST	10 × 12 deg	7.5	5 Hz	45 deg
Clyd. ST-200	-	-	5 Hz	45/30 deg
Clyd. ST-400	-	-	5 Hz	40 deg
Ku Leuv. ST	-	6	10 Hz	40 deg
TY NST-4	15 × 12 deg	5.8	10 Hz	25 deg
MAI-SS	-	6	4 Hz	90/45 deg
Sincl. ST-16	15 × 20 deg	-	2 Hz	34 deg
Tyvak (IRM)	16.8 × 12.6 deg	6	6 Hz	-

**Table 4:** M–ARGO NAVCAM characteristics.

Parameter	Value	Unit
Field of View	16 × 10	deg^2^
Image Size	2048 × 1280	pixels
Focal Length	40	mm
F-number	3.2	-
Aperture	12.5	mm
Bit depth	12	bits/pix

**Table 5:** Common image sensors for space applications.

		HAS2	FaintStar	CMV4000	LCMS
Parameter	Unit	Value	Value	Value	Value
Sensor Format	pix	1024 × 1024	1024 × 1024	2048 × 2048	512 × 512
Pixel Size	μm	18	10	5.5	25
ADC Res	bits	12	12	12	12
QE × FF	–	0.45	0.49	0.60	0.40
FWC	ke^−^	100	80	13.5	75
Quantization Noise	e^−^	7	6	3	6
Fixed-Pattern Noise	e^−^	115	44	13	20
Dark Signal	e^−^/s	190	174	125	1000
DSNU	e^−^/s	275	50	40	100
Read-Out Noise	e^−^	–	40	13	60
PRNU	–	0.018	–	0.010	0.015

**Table 6:** Values for SNR simulations.

Parameter	Unit	Value	Parameter	Unit	Value
Field of View	deg	16 × 10	QE × FF	–	0.50
Focal length	mm	40	FWC	ke^−^	100
F-number	–	3.2	Quantization Noise	e^−^	7
Aperture Diameter	mm	12.5	Fixed-Pattern Noise	e^−^	100
Sun Exclusion Angle	deg	35	Dark Signal	e^−^/s	200
Sensor Size	pix	2048 × 1280	DSNU	e^−^/s	100
Pixel Size	μm	5.5	Read-Out Noise	e^−^	100
Plate Scale	arcsec/pix	28.1250	PRNU	–	0.02
ADC Res	bits	12	Overall Noise Margin	%	20

**Table 7:** Estimated epochs of first detection for the M–ARGO targets.

Asteroid	Traject.	Arrival	Det. Epoch	Det. Epoch	Det. Epoch	Det. Epoch
			App Mag	SNR—1 s	SNR—10 s	SNR—100 s
		[MJD]	[MJD]	[MJD]	[MJD]	[MJD]
2000 SG344	Baseline	9545	9518	9518 (+0)	9512 (+06)	9511 (+07)
	Backup	9785	9769	9769 (+0)	9759 (+10)	9757 (+12)
2010 UE51	Baseline	9241	9240	9240 (+0)	9233 (+07)	9226 (+14)
	Backup	9887	9885	9885 (+0)	9878 (+07)	9871 (+14)
2011 MD	Baseline	9410	9402	9402 (+0)	9388 (+14)	9381 (+21)
	Backup	9612	9610	9610 (+0)	9603 (+07)	9596 (+14)
2012 UV136	Baseline	9517	9510	9510 (+0)	9502 (+08)	9495 (+15)
	Backup	9939	9930	9930 (+0)	9923 (+07)	9909 (+21)
2014 YD	Baseline	9329	9322	9322 (+0)	9308 (+14)	9296 (+26)
	Backup	9625	9617	9617 (+0)	9602 (+15)	9588 (+29)

**Table 8:** Settings for SNR sensitivity analysis: (1) low performance (LP), (2) reference performance (RP), and (3) high performance (HP).

Parameter	Unit	LP	RP	HP
F-number	–	4.0	3.2	2.6
QE × FF	–	0.45	0.50	0.55
Quantization Noise	e^−^	10	7	4
Aperture Diameter	mm	10	12.5	15
Fixed-Pattern Noise	e^−^	125	100	75
Sun Exclusion Angle	deg	40	35	30
Dark Signal	e^−^/s	250	200	150
Sensor Size	pix	1280 × 1280	2048 × 1280	2048 × 2048
DSNU	e^−^/s	150	100	50
Read-Out Noise	e^−^	150	100	50
PRNU	–	0.025	0.020	0.015
FWC	ke^−^	100	100	100
ADC Res	bits	12	12	12
Overall Noise Margin	%	25	20	15

## Data Availability

Not applicable.
